# Are public health researchers designing for dissemination? Findings from a national survey in China

**DOI:** 10.1186/s43058-023-00451-1

**Published:** 2023-09-05

**Authors:** Yiluan Hu, Xuejun Yin, Enying Gong, Jing Liu, Xia Liu, Ruitai Shao, Juan Zhang, Ross C Brownson

**Affiliations:** 1 School of Population Medicine and Public Health, Chinese Academy of Medical Sciences and Peking Union Medical College, No.9 Dong Dan San Tiao, Dongcheng District, Beijing, 100730 China; 2 The George Institute for Global Health, University of New South Wales, Newtown, NSW Australia; 3 Chinese Preventive Medicine Association, Beijing, 100021 China; 4 Research Unit of Population Health, Faculty of Medicine, University of Oulu, 5000 Oulu, Finland; 5 Prevention Research Center, Brown School at Washington University in St. Louis, One Brookings Drive, Campus, Box 1196, St. Louis, MO 63130 USA; 6 Department of Surgery, Division of Public Health Sciences, and Alvin J. Siteman Cancer Center, Washington University School of Medicine, Washington University in St. Louis, St. Louis, MO 63130 USA

**Keywords:** Dissemination, Designing for dissemination, China, Researchers

## Abstract

**Background:**

Research findings are not always disseminated in ways preferred by audiences, and research dissemination is not always considered a priority by researchers. While designing for dissemination (D4D) provides an active process to facilitate effective dissemination, use of these practices in China is largely unknown. We aimed to describe the designing for dissemination activities and practices among public health researchers in China.

**Methods:**

In January 2022, we conducted a cross-sectional survey in 61 sub-committees of four national academic societies which include a wide range of health disciplines. The sample mainly involved researchers at universities or research institutions, the Centers for Disease Control and Prevention at national or regional levels, and hospitals. Participants completed a 42-item online questionnaire. Respondent characteristics, dissemination routes, dissemination barriers, organizational support, and personal practice of D4D were examined with descriptive analyses.

**Results:**

Of 956 respondents, 737 were researchers. Among these researchers, 58.1% had disseminated their research findings. Although there were some variation in the commonly used routes among different groups, academic journals (82.2%) and academic conferences (73.4%) were the most frequently used routes. Barriers to dissemination to non-research audiences existed at both organizational level (e.g., a lack of financial resources, platforms, and collaboration mechanisms) and individual level (e.g., a lack of time, knowledge, and skills, and uncertainty on how to disseminate). About a quarter of respondents (26.7%) had a dedicated person or team for dissemination in their unit or organization, with university researchers reporting a significantly higher proportion than their counterparts (*P* < 0.05). Only 14.2% of respondents always or usually used frameworks or theories to plan dissemination activities, 26.2% planned dissemination activities early, and 27.1% always or usually involved stakeholders in the research and dissemination process. Respondents with working experience in a practice or policy setting or dissemination and implementation training experience were more likely to apply these D4D strategies (*P* < 0.05).

**Conclusion:**

Considerable room exists for improvement in using impactful dissemination routes, tackling multiple barriers, providing organizational support, and applying D4D strategies among Chinese public health researchers. Our findings have implications for structural changes in academic incentive systems, collaborations and partnerships, funding priorities, and training opportunities.

**Supplementary Information:**

The online version contains supplementary material available at 10.1186/s43058-023-00451-1.

Contributions to the literature

• Designing for dissemination (D4D) is an active process to enhance the uptake of research findings into routine clinical practice. Researches have shown considerable room for improvement in the use of D4D strategies and the provision of resources and support for D4D in other countries.
• To our knowledge, this study is the first to explore D4D in China, providing a baseline for future research, practice, and policy.
• The findings of this study suggest gaps in D4D in China and provide implications for structural changes in academic incentive systems, collaborations and partnerships, funding priorities, and training opportunities.

## Introduction

Dissemination, an active approach of spreading evidence-based interventions to the target audience via predetermined channels using planned strategies [1, 2], is the critical process linking research evidence to practitioners. Dissemination is insufficient globally [3, 4]. The disconnect between how researchers disseminate their findings and how practitioners and policymakers learn about and use them is a widely acknowledged chasm [5], and too-often, neither researchers nor practitioners assume the responsibility for dissemination activities despite the consensus on the importance of active dissemination [1, 6]. Designing for dissemination (D4D) has been recommended as an active process to ensure that public health and clinical interventions, often evaluated by researchers, are developed in ways that match well with adopters’ needs, assets, and time frames [1]. The D4D perspective highlights researchers’ responsibility for actively planning dissemination activities at the outset of a research, with the aim of enhancing the potential for adoption, sustainability, and ultimately impact of health and health equity [7].

Previous studies had explored the practice of D4D among researchers in the UK, US, Brazil, and Canada [1, 8–10]. In 2010, Wilson et al. systematically reviewed dissemination planning frameworks and strategies and invited public health researchers across the UK through funding agencies to report their dissemination activities [8, 11], which informed similar studies in the US and Brazil in 2012. In the US, public health researchers were selected from high-impact journals [1], while in Brazil, researchers in health sciences were drawn from the database available at the Brazilian Council for Scientific and Technological Development [9]. In 2018, given the development of Dissemination and Implementation (D&I) science and the trend of encouraging dissemination, an updated and expanded survey of the 2012 survey in the US was conducted among US and Canadian health researchers [10]. Results of these four surveys showed similarities in recognition of the importance of dissemination, the insufficient practice of D4D, and limited support and resources; while simultaneously observed apparent differences because of diverse contexts across countries.

D&I science is an emerging field in China. Although China has made great progress in evidence production over the past 20 years, with the number of academic publications increasing tenfold from 2000 to 2019 [12], disseminating and implementing evidence-based practices is complex and challengeable in China with large population, high burden of diseases, and extreme shortage of healthcare providers [13]. For example, universities—the most important producers of evidence in China [12]—are mainly concerned with publishing research findings in academic journals, which tends to be passive and unlikely to reach government officials and practitioners [14, 15]. Under the health system reforms and the “Healthy China” national strategy, the Chinese government has issued a series of policies to improve health for all. In this context, dissemination and implementation from research to practice has gained rising attention in recent years. Though progress has been made, dissemination in China is still in its infancy. Unlike UK, US, and Canada, where funding agencies have increasingly adopted policies to support or require dissemination and implementation [7, 8, 16–18], funding for dissemination in China is limited.

Although several studies have identified some determinants of evidence-practice gap from the perspectives of researchers, practitioners, and policy makers [19–23], D4D in China remains largely unknown. This cross-sectional study aimed to describe the dissemination activities and the practice of D4D among public health researchers in China, and to see how it may differ by researcher characteristics.

## Methods

### Design

A cross-sectional survey was conducted in January 2022 with four national academic societies in health areas in China after obtaining approval from the Chinese Academy of Medical Sciences and Peking Union Medical College Ethics Committee (CAMS&PUMC-IEC-2021–12). We used STrengthening the Reporting of OBservational studies in Epidemiology (STROBE) guideline to report the study (see Additional file 1).

### Survey instrument

The instrument was adapted from previous studies [1, 10] to fit the context in China. Initially, we interviewed 17 public health researchers to explore the context of dissemination in China. We found that some respondents mixed the concepts of disseminating their research findings with providing general health education to the public, which was more common among researchers in early stages of their career. According to the understanding of dissemination by respondents, we accurately translated “dissemination” into Chinese and provided background information in the preface of questionnaire to help participants understand the concept of dissemination. In China, academic societies usually are a group of researchers and practitioners, and our study subjects are researchers given that the D4D perspective highlights researchers’ responsibility for dissemination. Hence, we added an item in the questionnaire to identify those who conduct research by asking the question “Are you engaged in conducting research?” and those who answered “I am not engaged in conducting research” to the question completed the survey here. We also added an item to identify researchers who disseminated their research findings by asking the question “Have you ever disseminated your research findings?” and those who answered “no” to this question completed the survey here, while those who answered “yes” were defined as researchers who disseminated their research findings and continuingly answered questions about how they disseminated. The adapted questionnaire was further modified by consulting our advisory group members and cognitive response testing. The cognitive response testing was conducted in November 2021 with eight senior public health researchers to identify questions and concepts not easily comprehended, recalled, or with problematic response choices [24, 25]. An additional file shows the cognitive response testing interview guide (see Additional file 2). A pilot survey was run among a convenience sample of 12 public health researchers in Chinese Preventive Medicine Association (CPMA), who were similar to the respondents of our formal survey, to further verify the questionnaire and test the distribution channels.

The final version of the questionnaire (with 42 questions) (see Additional file 3) was used to gather data from respondents about their research information, dissemination activities, and practices of D4D: (1) characteristics of respondents: included questions about work place, highest attainment of education, years of conducting research, number of publications in peer-reviewed journals either as a first author or corresponding author in the recent three years, research settings, experience of working in a practice or policy setting where research might apply, and D&I training experience; (2) dissemination routes: three multiple choice questions were used to rate the most commonly used routes for dissemination, the most impactful routes on career trajectory, and the most impactful routes on public health practice or policy, respectively; (3) barriers to dissemination: a multiple choice question was used to identify what hindered researchers to disseminate to non-research audiences; (4) organizational supports for dissemination: included questions about support from employer and funding agencies; and (5) personal practices related to D4D (e.g., planning dissemination activities, producing summaries for non-research audiences, engaging stakeholders).

### Survey sample

Initially, respondents were invited by CPMA, the only organization that brings together a broad-based community of researchers and practitioners across a wide range of health disciplines in 91 professional sub-committees to improve the people’s health in China. With the exclusion of 18 sub-committees not relevant to research according to expert consultation, invitations were sent to 73 sub-committees. A total of 58 sub-committees (79.5%) agreed to participate in the study. Given that public health researchers in the field of nutrition, TB, and HIV/AIDS are members of Chinese Nutrition Society, Chinese Antituberculosis Association, and Chinese Association of STD and AIDS Prevention and Control, we recruited researchers in these fields from their respective sub-committees to supplement the disciplines not included in sub-committees of CPMA. Given that dissemination is a relatively new concept in China and dissemination practice might not be common among early career researchers, we only invited standing committee members who were senior researchers among their peers to constitute the sample. Inclusion criteria were (1) the secretary-general of the sub-committee agreed to participate in the study and facilitate the collection of the data from the standing committee members, and (2) standing committee members agreed to file the online questionnaire.

### Data collection

In January 2022, the secretaries-general of 58 sub-committees in CPMA sent the online questionnaire through its own questionnaire management platform with which the members were familiar. The secretaries-general in the other three academic societies sent it through Wenjuanxing (one of the biggest platforms to design electronic questionnaires in China) through WeChat (Chinese social software, similar to WhatsApp and Snapchat) groups; they sent a reminder message a week later to increase the response rate. A total of 956 respondents from approximately 1,466 members of the standing committee in 61 sub-committees were recruited, with a response rate of 65.2%.

### Statistical analysis

Data processing and database establishment were completed by Excel version 2016, and statistical analysis was performed in IBM SPSS version 25. No data was missing. Respondents’ characteristics, dissemination routes, dissemination barriers, organizational support, and personal practice of D4D were examined with descriptive analysis. The two continuous variables (i.e., years of conducting research, number of publications in peer-reviewed journals either as a first author or corresponding author in the recent three years) were transformed into categorical variables and presented as frequency and percent, along with all other categorical variables. Chi-square test was used for subgroup comparison. A *P* value of < 0.05 (two-sided) was considered statistically significant.

## Results

Of the 956 respondents, 219 were engaged in non-research work (e.g., policy making, public health practice, clinical practice) and were excluded from the current analyses. Among 737 researchers, 309 respondents did not disseminate their research findings, and 428 researchers (58.1%) did so.

### Characteristics of respondents

The majority of respondents worked in universities or research institutions (33.4%), followed by hospitals (32.4%), Centers for Disease Control and Prevention (CDC) at the national or regional level (30.4%), and a variety of other settings (3.8%) (Table 1). Among 428 researchers who disseminated their research findings, 34.3% worked in universities or research institutions, 31.8% in the CDC at the national or regional level, and 29.0% in hospitals. The research settings, where respondents conducted research, were mainly community settings (60.0%), clinical settings (40.7%), and laboratory settings (29.4%). The majority of respondents (63.1%) had previously worked in a practice or policy setting where their research findings might apply; 39.0% had received formal training in D&I. The highest percentage of those with previous experience of working in a practice or policy setting was that for researchers working in the CDC at national or regional level (76.5%); the highest of those with D&I training experience was that for researchers working in universities or research institutions (52.4%).

**Table 1:** Characteristics of researchers

	**Total (*n* = 737)**	**Researchers who did not disseminate (*n* = 309)**	**Researchers who disseminated (*n* = 428) **^**a**^
**Work place**			
University/research institution	246 (33.4)	99 (32.0)	147 (34.3)
CDC at national or regional level	224 (30.4)	88 (28.5)	136 (31.8)
Hospital	239 (32.4)	115 (37.2)	124 (29.0)
Other	28 (3.8)	7 (2.3)	21 (4.9)
**Highest attainment of academic degree**			
Doctorate	407 (55.2)	160 (51.8)	247 (57.7)
Master’s	223 (30.3)	100 (32.4)	123 (28.7)
Bachelor’s	106 (14.4)	48 (15.5)	58 (13.6)
Other	1 (0.1)	1 (0.3)	0
**Years of doing research**			
≤ 10 years	89 (12.1)	45 (14.6)	44 (10.3)
11–20 years	227 (30.8)	114 (36.9)	113 (26.4)
21–30 years	263 (35.7)	105 (34.0)	158 (36.9)
≥ 30 years	158 (21.4)	45 (14.6)	113 (26.4)
**Number of publications in peer-reviewed journals in the recent three years**			
0–2	164 (22.3)	95 (30.7)	69 (16.1)
3–5	200 (27.1)	94 (30.4)	106 (24.8)
6–11	180 (24.4)	61 (19.7)	119 (27.8)
≥ 12	193 (26.2)	59 (19.1)	134 (31.3)
**Research setting**			
Community	383 (52.0)	126 (40.8)	257 (60.0)
Clinical	303 (41.1)	129 (41.7)	174 (40.7)
Health Systems	121 (16.4)	40 (12.9)	81 (18.9)
Policy	105 (14.2)	34 (11.0)	71 (16.6)
Laboratory	231 (31.3)	105 (34.0)	126 (29.4)
Other	12 (1.6)	6 (1.9)	6 (1.4)
**Experience of working in a practice or policy setting**			
Yes	342 (46.4)	72 (23.3)	270 (63.1)
No	395 (53.6)	237 (76.7)	158 (36.9)
**D&I training experience**			
Yes	241 (32.7)	74 (23.9)	167 (39.0)
No	496 (67.3)	235 (76.1)	261 (61.0)

^a ^Subsequent analysis was based on this sample of researchers who had disseminated their research findings (*n* = 428). Abbreviations: *CDC* Centers for Disease Control and Prevention, *D&I* dissemination and implementation

### Routes for dissemination

Table 2 displays the dissemination routes utilized by respondents. Academic journals (82.2%) and academic conferences (73.4%) were the most frequently used routes for dissemination; while 23.8% reported face-to-face meetings with stakeholders. The top four impactful routes on personal career trajectory were academic journals (75.5%), academic conferences (46.0%), standards or guidelines (29.2%), and policy briefs (24.8%). Comparably, the top four impactful routes on public health practice or policy were policy briefs (56.3%), academic journals (45.6%), standards or guidelines (38.8%), and academic conferences (23.6%).

**Table 2:** Dissemination routes and impact

Routes	Commonly used		Most impact on career trajectory		Most impact on practice/policy	
	n (%)	ranking	n (%)	ranking	n (%)	ranking
Academic journals	352 (82.2)	1	323 (75.5)	1	195 (45.6)	2
Academic conferences	314 (73.4)	2	197 (46.0)	2	101 (23.6)	4
Reports to funders	177 (41.4)	3	57 (13.3)	5	44 (10.3)	8
Seminars/workshops	170 (39.7)	4	57 (13.3)	5	39 (9.1)	9
Policy briefs	163 (38.1)	5	106 (24.8)	4	241 (56.3)	1
Standards/guidelines	163 (38.1)	5	125 (29.2)	3	166 (38.8)	3
Newsletter/Reports to employers	144 (33.6)	7	32 (7.5)	8	30 (7.0)	10
Patent	109 (25.5)	8	30 (7.0)	10	18 (4.2)	11
Face-to-face meetings with stakeholders	102 (23.8)	9	39 (9.1)	7	48 (11.2)	7
Media interviews/Press releases	78 (18.2)	10	21 (4.9)	11	71 (16.6)	5
New media	76 (17.8)	11	32 (7.5)	8	52 (12.1)	6
Targeted mailings	21 (4.9)	12	1 (0.2)	12	1 (0.2)	12

The dissemination routes among respondents differed by work places, research settings, and previous experience of working in a practice or policy setting (Table 3). Researchers working in universities or research institutions or having previously worked in a practice or policy setting were more likely to use a variety of routes to disseminate (i.e., reports to funders, policy briefs, face-to-face meetings with stakeholders, and media interviews or press releases). However, there was no significant difference in dissemination routes by training experience in D&I.

**Table 3:** Commonly used routes for dissemination by work places, research settings, and experience of working in a practice or policy setting

Dissemination routes	Work place ^a^				Research setting				Experience of working in a practice/policy setting	
	**University/research institution (*n* = 147)**	**CDC at national or regional level (*n* = 136)**	**Hospital (*n* = 124)**	**Other (*n* = 21)**	**Community**		**Clinical**		**Yes (*n* = 270)**	**No (*n* = 158)**
					**Yes (*n* = 257)**	**No (*n* = 171)**	**Yes (*n* = 174)**	**No (*n* = 254)**		
Academic journals	118 (80.3)	120 (88.2)	101 (81.5)	13 (61.9)	219 (85.2)	133 (77.8)	144 (82.8)	208 (81.9)	227 (84.1)	125 (79.1)
Academic conferences	107 (72.8)	99 (72.8)	96 (77.4)	12 (57.1)	187 (72.8)	127 (74.3)	134 (77.0)	180 (70.9)	201 (74.4)	113 (71.5)
Reports to funders	73 (49.7)	57 (41.9)	40 (32.3)*	7 (33.3)	122 (47.5)	55 (32.2)*	66 (37.9)	111 (43.7)	124 (45.9)	53 (33.5)*
Seminars/workshops	63 (42.9)	52 (38.2)	46 (37.1)	9 (42.9)	104 (40.5)	66 (38.6)	72 (41.4)	98 (38.6)	110 (40.7)	60 (38.0)
Policy briefs	65 (44.2)	72 (52.9)	20 (16.1)*	6 (28.6)	135 (52.5)	28 (16.4)*	37 (21.3)	126 (49.6)*	120 (44.4)	43 (27.2)*
Standards/guidelines	52 (35.4)	57 (41.9)	44 (35.5)	10 (47.6)	104 (40.5)	59 (34.5)	64 (36.8)	99 (39.0)	116 (43.0)	47 (29.7)*
Newsletter/Reports to employers	42 (28.6)	60 (44.1)	33 (26.6)*	9 (42.9)	93 (36.2)	51 (29.8)	37 (29.4)	90 (35.4)	101 (37.4)	43 (27.2)*
Patent	50 (34.0)	26 (19.1)	30 (24.2)*	3 (14.3)	53 (20.6)	56 (32.7)*	46 (26.4)	63 (24.8)	69 (25.6)	40 (25.3)
Face-to-face meetings with stakeholders	43 (29.3)	34 (25.0)	18 (14.5)*	7 (33.3)	68 (26.5)	34 (19.9)	34 (19.5)	68 (26.8)	73 (27.0)	29 (18.4)*
Media interviews/Press releases	36 (24.5)	26 (19.1)	15 (12.1)*	1 (4.8)	54 (21.0)	24 (14.0)	26 (14.9)	52 (20.5)	59 (21.9)	19 (12.0)*
New media	29 (19.7)	28 (20.6)	17 (13.7)	2 (9.5)	55 (21.4)	21 (12.3)*	30 (17.2)	46 (18.1)	54 (20.0)	22 (13.9)
Targeted mailings	7 (4.8)	8 (5.9)	6 (4.8)	0	15 (5.8)	6 (3.5)	7 (4.0)	14 (5.5)	14 (5.2)	7 (4.4)

^*^P < 0.05

^a ^Excluded “Other” category when performed Pearson χ^2 ^test. Abbreviation: *CDC* Centers for Disease Control and Prevention

### Barriers to dissemination

Table 4 reveals that barriers to dissemination to non-research audiences existed at both organizational and individual levels. Organizational-level barriers included lack of financial resources (42.5%), platforms (41.1%), collaboration mechanisms (32.5%), incentives (22.0%), and relationships with stakeholders (18.0%), as well as dissemination activities not in study timelines (15.7%). At the individual level, lack of time (48.1%) and uncertainty on how to disseminate beyond professional conferences or publications (39.0%) were identified as the main barriers, followed by lack of knowledge and skills (26.9%), as well as uncertainty about audience make-up (22.4%), impact (21.0%) and content (15.4%).

**Table 4:** Barriers to dissemination to non-research audiences by work places, research settings, and experience of working in a practice or policy setting

Barriers	Total (*n* = 428)	Work place ^a^				Research setting				Experience of working in a practice/policy setting	
		**University/research institution** **(*n* = 147)**	**CDC at national or regional level (*n* = 136)**	**Hospital (n = 124)**	**Other (*n* =21 )**	**Community**		**Clinical**		**Yes (*n* = 270)**	**No (*n* = 158)**
						**Yes (*n* = 257)**	**No (*n* = 171)**	**Yes (*n* = 174)**	**No (*n* = 254)**		
**Organizational level**											
Lack of financial resources for dissemination	182 (42.5)	68 (46.3)	57 (41.9)	52 (41.9)	5 (23.8)	115 (44.7)	67 (39.2)	73 (42.0)	109 (42.9)	116 (43.0)	66 (41.8)
Lack of platforms for dissemination	176 (41.1)	55 (37.4)	62 (45.6)	49 (39.5)	10 (47.6)	110 (42.8)	66 (38.6)	71 (40.8)	105 (41.3)	104 (38.5)	72 (45.6)
Lack of collaboration mechanisms	139 (32.5)	43 (29.3)	50 (36.8)	38 (30.6)	8 (38.1)	93 (36.2)	46 (26.9)*	50 (28.7)	89 (35.0)	93 (34.4)	46 (29.1)
Lack of incentives	94 (22.0)	41 (27.9)	27 (19.9)	21 (16.9)	5 (23.8)	67 (26.1)	27 (15.8)*	35 (20.1)	59 (23.2)	58 (21.5)	36 (22.8)
Lack of relationships with stakeholders	77 (18.0)	27 (18.4)	26 (19.1)	17 (13.7)	7 (33.3)	52 (20.2)	25 (14.6)	18 (10.3)	59 (23.2)*	48 (17.8)	29 (18.4)
Dissemination activities not in study timelines	67 (15.7)	19 (12.9)	26 (19.1)	18 (14.5)	4 (19.0)	46 (17.9)	21 (12.3)	26 (14.9)	41 (16.1)	47 (17.4)	20 (12.7)
**Individual level**											
Lack of time	206 (48.1)	74 (50.3)	58 (42.6)	68 (54.8)	6 (28.6)	122 (47.5)	84 (49.1)	95 (54.6)	111 (43.7)*	129 (47.8)	77 (48.7)
Uncertainty on how to disseminate beyond professional conferences/publications	167 (39.0)	70 (47.6)	39 (28.7)	54 (43.5)*	4 (19.0)	91 (35.4)	76 (44.4)	78 (44.8)	89 (35.0)*	93 (34.4)	74 (46.8)*
Lack of knowledge and skills to disseminate	115 (26.9)	34 (23.1)	41 (30.1)	36 (29.0)	4 (19.0)	69 (26.8)	46 (26.9)	51 (29.3)	64 (25.2)	79 (29.3)	36 (22.8)
Uncertainty about audience make-up	96 (22.4)	34 (23.1)	19 (14.0)	41 (33.1)*	2 (9.5)	44 (17.1)	52 (30.4)*	53 (30.5)	43 (16.9)*	49 (18.1)	47 (29.7)*
Uncertainty about the impact of dissemination	90 (21.0)	21 (14.3)	31 (22.8)	34 (27.4)*	4 (19.0)	52 (20.2)	38 (22.2)	42 (24.1)	48 (18.9)	55 (20.4)	35 (22.2)
Uncertainty about what to disseminate	66 (15.4)	20 (13.6)	17 (12.5)	28 (22.6)	1 (4.8)	37 (14.4)	29 (17.0)	34 (19.5)	32 (12.6)	35 (13.0)	31 (19.6)
Hesitation/resistance to disseminate findings from a single study	39 (9.1)	8 (5.4)	20 (14.7)	9 (7.3)*	2 (9.5)	27 (10.5)	12 (7.0)	13 (7.5)	26 (10.2)	25 (9.3)	14 (8.9)

^*^P < 0.05

^a ^Excluded “Other” category when performed Pearson χ^2 ^test. Abbreviation: *CDC* Centers for Disease Control and Prevention

Respondents’ perception of organizational-level barriers differed by research settings, yet showed no difference by work places and previous experience of working in a practice or policy setting (Table 4). On the other hand, individual-level barriers differed by work places, research settings, and previous experience of working in a practice or policy setting. However, there was no variation at both organizational- and individual-level barriers by training experience in D&I.

### Organizational support

Table 5 presents the findings of organizational support from employers and funding agencies. Although the majority of respondents reported dissemination to non-research audiences was expected by their employers (75.9%), 26.7% reported there was a dedicated person or team for dissemination and 25.7% reported a formal dissemination strategy or plan in their unit or organization. Those dedicated persons or teams were mainly housed within the Office of Translation (31.6%), the Communication Office (21.9%), the General Office (17.5%), and the Development Office (14.3%). More researchers working in universities or research institutions reported a dedicated person or team (32.0% vs. 16.9%) and a formal strategy or plan (34.0% vs. 15.4%) in their unit or organization, than their counterparts in the CDC at national or regional level.

**Table 5:** Organizational support for designing for dissemination by work places

**Support**	**Total (*n* = 428)**	**University/research institution (*n* = 147)**	**CDC at national or regional level (*n* = 136)**	**Hospital (*n* = 124)**	**Other (*n* = 21)**	${{\varvec{\chi}}}^{2}$	*P *^**a**^
**From employers**							
**Dissemination to non-research audiences is expected by department **^**b**^						1.093	0.579
Yes	325 (75.9)	114 (77.6)	105 (77.2)	90 (72.6)	16 (76.2)		
No	11 (2.6)	5 (3.4)	3 (2.2)	3 (2.4)	0		
Not sure	92 (21.5)	28 (19.0)	28 (20.6)	31 (25.0)	5 (23.8)		
**Dedicated person/team responsible for dissemination in unit/organization**						11.419	0.076
Yes	114 (26.6)	47 (32.0)	23 (16.9)	37 (29.8)	7 (33.3)		
No	224 (52.3)	69 (46.9)	85 (62.5)	61 (49.2)	9 (42.9)		
Not sure	90 (21.0)	31 (21.1)	28 (20.6)	26 (21.0)	5 (23.8)		
**Formal dissemination strategy or plan in unit/organization**						16.195	0.013
Yes	110 (25.7)	50 (34.0)	21 (15.4)	35 (28.2)	4 (19.0)		
No	164 (38.3)	50 (34.0)	65 (47.8)	41 (33.1)	8 (38.1)		
Not sure	154 (36.0)	47 (32.0)	50 (36.8)	48 (38.7)	9 (42.9)		
**From funding agencies**							
**Dissemination to non-research audiences is expected by funding agencies **^**b**^						7.438	0.282
Yes	254 (59.3)	91 (61.9)	74 (54.4)	76 (61.3)	13 (61.9)		
No	27 (6.3)	13 (8.8)	5 (3.7)	8 (6.5)	1 (4.8)		
Not sure	147 (34.3)	43 (29.3)	57 (41.9)	40 (32.3)	7 (33.3)		
**Funding support various dissemination activities**						6.415	0.040
Yes	147 (34.3)	61 (41.5)	37 (27.2)	42 (33.9)	7 (33.3)		
No	281 (65.7)	86 (58.5)	99 (72.8)	82 (66.1)	14 (66.7)		

^a ^Excluded “Other” category when performed Pearson χ^2 ^test

^b ^Grouped “No” category and “Not sure” category when performed Pearson χ^2 ^test. Abbreviation: *CDC* Centers for Disease Control and Prevention

Likewise, although 59.3% of respondents reported dissemination to non-research audiences was expected by their funding agencies, 65.7% reported the funding merely supporting academic publications or conferences, while 34.3% reported it supporting various dissemination activities (Table 5). Researchers working in universities or research institutions were more likely to report it supporting various dissemination activities (41.5%), while 27.2% researchers working in the CDC at national or regional level reported so.

### Personal practice of designing for dissemination

Table 6 shows several important activities related to D4D. Although 70.6% had ever used frameworks or theories to plan dissemination activities, as low as 14.2% reported always or usually did so. Additionally, among those who reported doing so, 59.1% did not recall or describe frameworks or theories they used. Respondents usually planned dissemination activities after results have been published or presented at academic meetings (58.5%), followed by at the moment of developing draft report or manuscript (28.1%), developing proposal (26.2%), implementation or data collection (13.9%), and data analysis (9.2%).

**Table 6:** Personal practice of designing for dissemination by work places, research settings, and experience of working in a practice or policy setting

Dissemination practice	Total (*n* = 428)	Experience of working in a practice/policy setting		D&I training experience	
		**Yes (*n* = 270)**	**No (*n* = 158)**	**Yes (*n* = 167)**	**No (*n* = 261)**
**Use of framework/ theory to plan dissemination activities (n = 381) **^a^					
Always/Usually	54 (14.2)	43 (17.7)	11 (8.0)	31 (19.5)	23 (10.4)
Sometimes/rarely	215 (56.4)	130 (53.5)	85 (61.6)	95 (59.7)	120 (54.1)
Never	61 (16.0)	39 (16.0)	22 (15.9)	14 (8.8)	47 (21.2)
Not sure	51 (13.4)	31 (12.8)	20 (14.5)	19 (11.9)	32 (14.4)*
**How often produce summaries for non-research audiences**					
Always/Usually	47 (11.0)	36 (13.3)	11 (7.0)	24 (14.4)	23 (8.8)
Sometimes/rarely	279 (65.2)	180 (66.7)	99 (62.7)	114 (68.3)	165 (63.2)
Never	54 (12.6)	25 (9.3)	29 (18.4)	13 (7.8)	41 (15.7)
Not sure	48 (11.2)	29 (10.7)	19 (12.0)*	16 (9.6)	32 (12.3)*
**How often stakeholders involved**					
Always/Usually	116 (27.1)	91 (33.7)	25 (15.8)	53 (31.7)	63 (24.1)
Sometimes/rarely	235 (54.9)	146 (54.1)	89 (56.3)	93 (55.7)	142 (54.4)
Never	41 (9.6)	16 (5.9)	25 (15.8)	9 (5.4)	32 (12.3)
Not sure	36 (8.4)	17 (6.3)	19 (12.0)*	12 (7.2)	24 (9.2)
**How often the uptake of research are evaluated**					
Always/Usually	61 (14.3)	45 (16.7)	16 (10.1)	32 (19.2)	29 (11.1)
Sometimes/rarely	274 (64.0)	173 (64.1)	101 (63.9)	112 (67.1)	162 (62.1)
Never	42 (9.8)	23 (8.5)	19 (12.0)	6 (3.6)	36 (13.8)
Not sure	51 (11.9)	29 (10.7)	22 (13.9)	17 (10.2)	34 (13.0)*

^*^P < 0.05

^a ^Respondents who did not plan dissemination activities were excluded for this question (*n* = 47). Abbreviation: *D&I* dissemination and implementation

Similarly, although most respondents reported they had ever produced summaries for non-research audiences (76.2%), involved stakeholders in conducting research and disseminating research findings (82.0%), and evaluated the uptake of research on public health practice or policy (78.3%), the proportion of always or usually conducting these activities were 11.0%, 27.1%, and 14.3%, respectively (Table 6).

Several important activities related to D4D showed differences by experience of working in a practice or policy setting, and D&I training experience, yet no differences were observed by work places and research settings (Table 6). Respondents with experience of working in a practice or policy setting were more likely to always or usually produce summaries for non-research audiences (13.3% vs. 7.0%, *P* < 0.05), and involve stakeholders in conducting research and disseminating research findings (33.7% vs. 15.8%, *P* < 0.05). Similarly, respondents with D&I training experience were more likely to always or usually use a framework or theory (19.5% vs. 10.4%, *P* < 0.05), produce summaries for non-research audiences (14.4% vs. 8.8%, *P* < 0.05), involve stakeholders (31.7% vs. 24.1%, *P* < 0.05), and evaluate the uptake of research (19.2% vs. 11.1%, *P* < 0.05).

## Discussion

To our knowledge, this is the first study to explore how Chinese public health researchers are following D4D principles. Our results suggest substantial room for D4D in China. The present study reported that only 58% of researchers were disseminating their research findings. Most of them disseminated through academic journals and academic conferences and were not D4D. Our findings provide implications for employers and funding agencies seeking to systematically change dissemination infrastructure and provide substantive support for dissemination to alleviate obstacles at the organizational and individual levels.

### Misalignment of dissemination routes and impact

Dissemination through academic journals (82%) and academic conferences (73%) among Chinese researchers remained dominant, despite that they believed other routes (e.g., policy briefs, standards or guidelines) to be more impactful on public health practice or policy. This was consistent with findings in the UK, US, and Canada [8, 10, 26]. Academic journals are less effective than other channels in reaching and engaging practitioners and policymakers due to limitations on access, time, resource reliability, or information overload [15, 19, 27–30]. A related study conducted by Choi et al. [19] reported that Chinese researchers and policymakers were less likely to cite academic journals as an ideal strategy to bridge the gap between science and policy than Canadian colleagues (23% vs. 43%). The misalignment of dissemination routes and impact is likely due in part to academic incentive systems focusing more on academic publications [10, 21, 31–33]. Our results that researchers perceived a high impact of academic journals on personal career also indicated the role of academic incentive systems. In addressing this issue, the Chinese government issued a policy [34] in 2020 encouraging researchers to disseminate research findings in many ways rather than only via academic publications. This paves the way for D4D to promote greater impact on health in China.

McVay et al. [26] reported that 68% of US researchers used face-to-face meetings with stakeholders to disseminate in 2012, and Knoepke et al. [10] reported that 55% of US and Canadian researchers used this route in 2019. However, in our study, Chinese researchers reported using this route less often (24%) and perceiving less impact of this route on practice or policy. This could be a COVID effect that researchers are less likely to meet in person these days. Instead, they preferred to disseminate through standards or guidelines. As informed by expert consultancy and cognitive response testing, many Chinese researchers are involved in developing standards and guidelines. Therefore, we included developing standards and guidelines in the response items of disseminating routes for research findings. Our findings suggest that over one-third of researchers disseminated research findings by developing standards or guidelines and emphasized the importance of this route on practice or policy. Standards and guidelines review and summarize the latest evidence and provide operable and unified recommendations for their use [35, 36]. Thus, the use of standards and guidelines shows promise for minimizing variability in clinical and public health practice in different settings across China [36, 37]. Despite the number of guidelines increasing annually, low methodological quality, the potential conflict of interest, and poor implementation status need to be addressed for improvements in healthcare and public health [37].

### Multiple barriers at the individual and organizational level

Evidence suggests that those who can make the biggest differences in improving population health are often non-researchers outside of the health professions (e.g., city planners, transportation officials, community-based organizations) [38]. However, barriers existing at the individual and organizational levels hinder the researchers from disseminating to non-research audiences. At the individual level, a lack of time, as well as inadequate capacity (including uncertainty on dissemination routes, a lack of knowledge and skills, uncertainty on dissemination audiences) were prominent barriers. This was generally consistent with previous findings among US researchers in 2012 [26]. An international comparison qualitative study suggested that limited time was a global phenomenon to prevent researchers from disseminating research findings rather than a barrier to higher-income countries only [20]; a review in low- and middle-income countries (LMICs) suggested that inadequate capacity was the major barrier to researchers’ dissemination practice [30]. Consistent with previous studies [13, 26, 30, 32], our finding suggested a lack of financial resources as one of the biggest and the most common barriers at the organizational level. Nevertheless, unlike the US colleagues who perceived a lack of staff time and academic incentives [26], Chinese researchers felt hindered by a lack of platforms and collaboration mechanisms. This might be a common finding across studies in LMICs [30].

We observed a high degree of variation at the individual-level barriers by work places, research settings, and work experiences, but less variation at the organization level. This might suggest that barriers are relatively consistent across organizations since dissemination of research findings is quite new in the Chinese context. In addition, researchers may know little about the opportunities or resources available in their organization, a better way to measure organizational-level barriers is needed. Given the trend of increasing training programs in D&I [10], we had assumed that researchers with D&I training experience would be less likely to perceive barriers than those without. However, there was no variation at both individual- and organizational-level barriers by D&I training experience. This may be due to that most D&I training programs focus more on how to conduct D&I research rather than on how to disseminate and implement. Furthermore, it is necessary to improve the quality of D&I training programs and expand the opportunities to participate in D&I training programs [39–42].

### Gaps between organizational expectation and support

The relationship between individuals and organizations is reciprocal: individuals shape organizations, and organizations support the development of individuals [5]. Ideally, organizational support would help overcome barriers and promote dissemination. For example, not everyone who perceived a lack of time or skills to write policy briefs needs to be trained in writing; instead, a person or team dedicated to dissemination (e.g., policy expert, communication expert) could lead efforts in writing briefs. There are multiple guidelines and templates for writing effective policy briefs [43–45].

Our study suggested a gap between organizational needs for D4D and support. Even though three-quarters of employers expected dissemination to non-research audiences, only a quarter of them had a person or team dedicated to dissemination. This was much lower than the US researchers who reported 53% had a person or team dedicated for dissemination [1]. To address this issue, the Chinese Research Hospital Association (one of the national academic societies that aim to integrate clinical practice and research) is promoting a Group Standard of Specification for Health Communication Practitioners [46] to support more systematic and professional dissemination. There was a similar chasm in funding agencies between the full expectation of dissemination and limited financial support for dissemination. Systematic-level changes in infrastructure are needed to support dissemination (e.g., involving transdisciplinary teams, shifting funding agencies’ priorities and processes, shifting researcher incentives and opportunities, developing new measures and tools) [1].

### Inadequate practice of designing for dissemination

Our findings showed the inadequate practice of D4D among Chinese researchers. For example, only 26% of respondents planned dissemination activities from the beginning of their study, and only 14% always or usually used a framework or theory to plan. These results were comparable to US participants in 2012 (27% and 17%, respectively) [1]. Only 27% of Chinese researchers always or usually involved stakeholders in their research progress, which was slightly lower than the US researchers in 2012 (34%) [1] and far from the US and Canadian researchers in 2018 (55% of respondents engaged stakeholders more than four times within a project) [10]. Chinese researchers also reported different methods of involving stakeholders from the US and Canadian researchers. This may be due in part to contextual differences—for example, stakeholder needs and engagement methods may be different in China [10]. Although involving many types of stakeholders (e.g., patients or consumers, the general public, practitioners, decision-makers, policymakers, funding agencies) at each stage in the research process is crucial [47], involving all of them in every process is unrealistic [1]. The degree and method of engagement should depend on stakeholders’ skills and types, as well as the capacity and needs of researchers [48].

The lack of attention to D4D in early phases of a project, the limited application of theories and frameworks, and the insufficient stakeholder involvement may influence the effective dissemination and implementation of health innovations [7]. Our findings suggested that researchers with working experience in a practice or policy setting and researchers with D&I training experience showed a better practice of D4D than those without. This was consistent with previous findings [1, 10], indicating the importance of involving researchers in practice settings, and building capacity in dissemination and implementation science.

### Limitations

We recognize some limitations to our study. First, the data were self-reported by researchers, which may differ from actual dissemination practice and organizational support. Second, we did not collect potential covariates such as sex/gender and sub-committee. This would not allow us to explore possible differences in the dissemination practices between male and female researchers, and among researchers from different disciplines. Third, dissemination is a new field, the interpretation may be not well-defined. We conducted qualitative interviews, cognitive response testing, and pilot testing to design a questionnaire that fits the Chinese context. Finally, the study sample was from the standing committees in academic societies, thus leading to selection bias and limiting the representativeness to all public health researchers in China. However, this study provides an initial exploration in China where D4D is in its infancy, and the insufficient dissemination efforts of standing committee members might indicate that dissemination by other researchers may be even more inadequate.

## Conclusion

Chinese public health researchers’ efforts in dissemination has room for improvement. Although researchers disseminated through various routes, they mainly focused on academic publications and academic conferences, yet rarely designed for dissemination. They faced barriers such as a lack of resources (e.g., financial support, collaboration mechanisms), incentives, and capacity. Structural changes in funder priorities, partnerships, academic incentive systems, and training opportunities are needed for more effective and efficient dissemination.

### Supplementary Information

**Additional file 1.** STROBE checklist for cross-sectional studies

**Additional file 2.** Cognitive Response Test Interview Guide.

**Additional file 3.** Questionnaire.

## Data Availability

All the data and materials of this study are available from the corresponding author on reasonable request.

## Abbreviations

D&I: Dissemination and Implementation
D4D: Designing for dissemination
STROBE: STrengthening the Reporting of OBservational studies in Epidemiology
CPMA: Chinese Preventive Medicine Association
CDC: Centers for Disease Control and Prevention
