# Supplementary material for: Are public health researchers designing for dissemination? Findings from a national survey in China
Source: Implement Sci Commun. 2023 Sep 5;4:110. doi: 10.1186/s43058-023-00451-1 (PMC10478366; doi:10.1186/s43058-023-00451-1)
Supplement: Supplementary file 2 — Additional file 2. Cognitive Response Test Interview Guide. [file 43058_2023_451_MOESM2_ESM.docx]

**访谈提纲**

**Cognitive Response Testing Interview Guide**

**一、 开场介绍**

**1. 项目及访谈内容介绍**

您好！感谢您百忙之中参加“科技工作者科研成果的推广”研究。本研究拟通过问卷调查，了解我国公共卫生研究人员推广自身科研成果的现状，为促进科研成果的推广与实施提供依据。在开展正式调查之前，我们诚挚邀请您参加我们的专家调查，对问卷的条目、选项设置的适用性和完整性，及语言表达、语义、作答方式是否符合中国的文化习惯等方面进行评价，我们将根据您的反馈进一步修改完善问卷。

本次访谈约1个小时，我们已事先邀请您填写问卷，请您根据您的感受谈谈问卷中哪些问题难以理解、难以回答或毫无意义，您所提供的任何信息都对我们意义重大。

**2. 知情同意**

为了便于后期信息的整理，我们希望能对今天讨论的内容录音。录音文件在文字化后会被删除，访谈的内容仅作学术分析，将来的文章和报告中不会体现出您的个人信息。

**Part A. Introduction**

**1. Introduction to the survey and interview**

Thank you for your participation in the survey of Disseminating the Findings of Public Health Research. Our aim is to understand the practice of dissemination of public health research findings among Chinese researchers, and hence, to improve the dissemination and implementation of research findings in China. Before conducting a large scale survey, we sincerely invite you to share your opinion on question comprehension, information retrieval and decision processing. Your input will help us improve our survey and ensure that the most useful information is collected.

The interview will take about one-hour to talk about what questions might be difficult to understand, hard to answer, or make little sense based on the questionnaire we have sent you early. Any information you provide is of interest, even it seems irrelevant or not important.

**2. Informed consent**

Your participation is confidential and will not be reported in a way that will identify you. With your permission, we would like to record our conversation to ensure your feedback is not overlooked.

**二、 前言部分**

1. 您认为前言部分对于您理解研究的背景有帮助吗？您还需要什么信息才能更好地理解研究的背景？

2. 您认为我们对于“推广”的定义翻译准确吗？您是如何理解这一定义的？

**Part B. Preface of the questionnaire**

1. Is the preface helpful in understanding the survey? What information do you need to better understand it?

2. Do you think our definition of “dissemination” has been translated accurately? What do you think the definition is talking?

**三、 基本信息部分**

本部分是一些常见问题（1-13题），旨在了解受试者的基本信息。

1. 您认为这些问题的选项合适吗？

2. 选项是否有重复或遗漏？

3. 是否还缺少一些关键问题？

**Part C. Background questions**

Question 1 to 13 are commonly used background questions.

1. Is there are any responses that don’t work in Chinese contexts?

2. Is there any response missing here?

3. Is there any questions missing here?

**四、 科研成果推广相关情况**

本部分旨在了解科研人员推广科研成果的情况（14-42题），如推广渠道、推广对象、阻碍因素、组织支持、个人实践等，请您对照下列问题谈谈您的任何建议或意见。

1. 您认为这个问题在问什么？

2. 这个问题容易回答吗？

3. 您需要什么信息才能更好地回答这个问题？

4. 您有什么建议帮助我们更好地改进这个问题吗？

5. 回答的选项有遗漏吗？

**Part D. Dissemination-related questions**

Question 14 to 42 aim to understand the practice of dissemination, such as dissemination methods, dissemination audiences, dissemination barriers, organizational support, personal practice, etc. Please read these questions carefully and provide any suggestions or opinions according to the following questions.

1. What do you think the question is asking?

2. Is this question hard or easy to answer?

3. What information do you need to answer the question?

4. Do you have suggestions on how to improve the question?

5. Is there a response that is missing here?

**五、 总结**

非常感谢您对我们的帮助和支持，您对我们的研究还有其他建议吗？

**Part E. Conclusion**

Thank you for completing this interview! Do you have any suggestions for this survey?
