# Supplementary material for: Are public health researchers designing for dissemination? Findings from a national survey in China
Source: Implement Sci Commun. 2023 Sep 5;4:110. doi: 10.1186/s43058-023-00451-1 (PMC10478366; doi:10.1186/s43058-023-00451-1)
Supplement: Supplementary file 3 — Additional file 3. Questionnaire. [file 43058_2023_451_MOESM3_ESM.docx]

**“科技工作者科研成果的推广”调查问卷**

**Disseminating the Findings of Public Health Research**

尊敬的女士/先生 ：

      您好！感谢您百忙之中参加“科技工作者科研成果的推广”研究。

      科研成果推广与实施性科学研究是近年来新发展起来的一类研究，是病因和发病机制研究、治疗和公共卫生干预措施研究基础上的一个新阶段，旨在促进循证基础上的治疗和公共卫生干预措施在真实世界的应用，弥合研究和实践之间的鸿沟，使科研成果促进人群健康方面尽快发挥起作用。

      本研究旨在了解科技工作者推广自身科研成果现状，为促进领域科研成果的推广与实施提供依据。

      本研究收集到的所有信息我们将严格保密，以后公布的调查结果和报告等也不会包含您的个人信息。如果您对本研究有任何疑问，可以与以下负责人联系：

      联系人：中华预防医学会 刘静

      电话：13810604806

      座机：010-64013358

      联系人: 中国医学科学院北京协和医学院群医学及公共卫生学院 张娟

      电话：13718177673

      如您同意参与本研究，请您按照要求填写问卷。如您不同意，您可以随时中断本次问卷调查。

Dear Madam/Sir,

      Thank you for your participation in the survey of Disseminating the Findings of Public Health Research.

      Over the past 20 years, the field of dissemination and implementation (D&I) science, which is a new stage based on efficacy research and effectiveness research, emerged as part of a collective commitment to accelerate and improve translation of evidence into practice.

     Our aim is to understand the practice of dissemination of public health research findings among Chinese researchers, and hence, to improve the dissemination and implementation of research findings in China.

      Your participation is confidential and will not be reported in a way that will identify you. If you have any questions, please contact:

      Jing Liu (Chinese Preventive Medicine Association) at 13810604806

      Juan Zhang (Chinese Academy of Medical Sciences and Peking Union Medical College) at 13718177673

      If you agree to participate in this study, please fill in the questionnaire as required. If you disagree, you can interrupt or quit at any time.

     本次调查中，我们将科研成果推广定义为：通过**主动、有计划**的方式和过程将科研成果更好地被他人和社会**了解和应用**。

      温馨提示：推广的内容是**您本人的科研成果**，而不是他人的科研成果或一般的健康知识。

      For this survey, we define dissemination as: An **active and planned** process that ensures that those who can use your research **learn about it and can make use of the findings**.

      Warm tip: dissemination is about **your own research research findings**, not others' research findings or general health knowledge.

1. 您的工作单位是？ [单选题] *

| ○高校 |
| --- |
| ○国家级科研机构 |
| ○省市一级科研机构 |
| ○中国疾病预防控制中心 |
| ○省、市、县级疾病预防控制机构 |
| ○学会/协会等学术团体组织 |
| ○国际组织 |
| ○医院 |
| ○其他，请注明 _________________ * |

1. Where do you work? [Single choice question] *

| ○University |
| --- |
| ○National research institution |
| ○Regional research institution |
| ○Chinese center for disease control and prevention |
| ○Regional center/agency for disease control and prevention |
| ○Academic society/association |
| ○International organization |
| ○Hospital |
| ○Other, please specify _________________ * |

2. 您的最高学位是？ [单选题] *

| ○博士学位 |
| --- |
| ○硕士学位 |
| ○学士学位 |
| ○其他，请注明 _________________ * |

2. What type is your highest academic degree? [Single choice question] *

| ○Doctorate |
| --- |
| ○Master’s |
| ○Bachelor’s |
| ○Other, please specify _________________ * |

3. 目前，您是从事科研工作还是其它工作？ [单选题] *

| ○我主要从事科研工作 (请跳至第5题) |
| --- |
| ○我大部分时间从事科研工作 (请跳至第5题) |
| ○我同时从事科研工作和其它工作 |
| ○我大部分时间从事非科研工作 |
| ○我主要从事非科研工作 (请跳至第问卷末尾，提交答卷) |

3. Are you engaged in conducting research? [Single choice question] *

| ○I am engaged in conducting research (Please skip to Question 5) |
| --- |
| ○I am mainly engaged in conducting research (Please skip to Question 5) |
| ○I am engaged in conducting research and non-research working |
| ○I am mainly engaged in non-research working |
| ○I am not engaged in conducting research (Please skip to the end of the questionnaire) |

4. 除科研工作外，您主要还从事以下哪类工作？ [单选题] *

| ○政策制定工作 |
| --- |
| ○公共卫生实践工作 |
| ○临床工作 |
| ○教学工作 |
| ○行政管理工作 |
| ○其他，请注明 _________________ * |

4. What kind of non-research working are you engaged in? [Single choice question] *

| ○Policy making |
| --- |
| ○public health practice |
| ○clinical |
| ○teaching |
| ○administrative |
| ○Other, please specify _________________ * |

5. 您在哪一年开始从事科研工作？ [下拉选择] *

5. In what year did you start doing research? [Pull-down listing years] *

6. 您目前的研究主要在什么领域？ [多选题] *

可多选，请选择所有符合的选项。

| □流行病学 |
| --- |
| □生物统计 |
| □环境卫生 |
| □职业卫生 |
| □营养与食品卫生 |
| □学校卫生 |
| □妇幼卫生 |
| □老年健康 |
| □卫生毒理 |
| □感染性疾病/传染性疾病 |
| □慢性非传染性疾病 |
| □行为健康/健康教育 |
| □全球健康 |
| □社会医学与卫生事业管理 |
| □卫生健康政策 |
| □其他，请注明 _________________* |

6. What are the academic areas of your research? [Multiple choice question] *

| Please select all that apply.  □Epidemiology |
| --- |
| □Biostatistics |
| □Environmental health |
| □Occupational health |
| □Nutrition |
| □School health |
| □Women and children health |
| □Health aging |
| □Hygienic toxicology |
| □Infectious diseases |
| □Non-communicable diseases |
| □Behavioral science |
| □Global health |
| □Social medicine and health service management |
| □Policy |
| □Other, please specify _________________* |

7. 您主要的研究场景是？ [多选题] *

可多选，请选择所有符合的选项。

| □人群（社区、学校、工作场所等） |
| --- |
| □临床（住院或门诊） |
| □医疗卫生服务体系 |
| □卫生健康政策 |
| □实验室 |
| □其他，请注明： _________________* |

7. In what setting do you do most of your research? [Multiple choice question] *

Please select all that apply.

| □Community settings |
| --- |
| □Clinical (inpatient or outpatient) |
| □Health delivery systems |
| □Policy settings |
| □Laboratory |
| □Other, please specify _________________* |

8. 近3年，您作为第一作者或通讯作者发表（见刊）的学术文章数量是？ [下拉选择] *

8. Number of publications in peer-reviewed journals either as a first author or corresponding author in the recent 3 years [Pull-down listing numbers] *

9. 您的研究经费来源有哪些？ [多选题] *

可多选，请选择所有符合的选项。

| □本单位的工作经费 |
| --- |
| □本单位的竞争性研究经费 |
| □横向委托经费 |
| □横向竞争性研究经费 |
| □纵向竞争性研究经费（如国自然） |
| □重大课题研究经费（如国家科技重大专项、国家重点研发经费等） |
| □国际竞争性研究经费 |
| □其他，请注明： _________________* |

9. What funding have you received? [Multiple choice question] *

Please select all that apply.

| □Working funds in my unit/organization |
| --- |
| □Competitive research funds in my unit/organization |
| □Entrusted funds |
| □Competitive commercial research funds |
| □Competitive public and government-sponsored research funds (e.g. National Natural Science Foundation of China, etc.) |
| □National key research funds (e.g. National Science and Technology Major Project, The National Key R&D Program of China, etc.) |
| □International competitive research funding |
| □Other, please specify _________________* |

10. 您获得的科研经费支持您怎样推广科研成果？ [单选题] *

| ○仅支持发表学术文章、参加学术会议、出版书籍等 |
| --- |
| ○除支持发表学术文章、参加学术会议、出版书籍等，还支持其他推广形式，请注明 ______________ * |

10. How do the funds you received support dissemination? [Single choice question] *

| ○merely support publishing academic publications or presenting in academic conferences |
| --- |
| ○support various dissemination activities other than merely publishing academic publications or presenting in academic conferences, please specify _________________ * |

11. 您是否接受过有关知识转化、推广性研究、实施性研究的系统培训？ [多选题] *

可多选，请选择所有符合的选项。

| □我参加过知识转化相关的系统培训 |
| --- |
| □我参加过推广性研究相关的系统培训 |
| □我参加过实施性研究相关的系统培训 |
| □我没有参加过这些相关的系统培训 |

11. Have you received formal post-doctoral training in knowledge translation or dissemination and implementation research? [Multiple choice question] *

Please select all that apply.

| □I have been trained in knowledge translation |
| --- |
| □I have been trained in dissemination research |
| □I have been trained in implementation research |
| □I have not been trained in these fields (Please skip to Question 13) |

12. 您参加过的上述培训是什么形式的？ [多选题] *

可多选，请选择所有符合的选项。

| □至少1年的长期国外访问学习 |
| --- |
| □至少1年的长期国内专题培训 |
| □至少1个季度/学期的大学课程 |
| □为期数天的短期课程 |
| □单日讲座 |
| □线上课程 |
| □其他，请注明： _________________* |

12. What format did this training take? [Multiple choice question] *

Please select all that apply.

| □Long-term training abroad for at least 1 year |
| --- |
| □Long-term domestic training for at least 1 year |
| □University course for at least a quarter or semester |
| □Multi-day short course |
| □Single day presentation |
| □Online course |
| □Other, please specify _________________* |

13. 您是否曾经在可以应用您的科研成果的工作场所或政策制定环境中工作过？ [单选题] *

| ○是 |
| --- |
| ○否 |
| ○不确定 |

13. Have you ever worked in a practice or policy setting where your research might be applicable? [Single choice question] *

| ○Yes |
| --- |
| ○No |
| ○Not sure |

14. 您是否推广过您的科研成果？ [单选题] *

| ○是 |
| --- |
| ○否 (请跳至第问卷末尾，提交答卷) |

14. Have you ever disseminated your research findings? [Single choice question] *

| ○Yes |
| --- |
| ○No (Please skip to the end of the questionnaire) |

15. 您一般通过哪些渠道推广您的科研成果？ [多选题] *

可多选，请选择所有符合的选项。

| □国内外学术期刊 |
| --- |
| □向经费支持机构提供报告 |
| □向所在单位提供报告或内部简讯 |
| □政策建议、报告、简报或专刊 |
| □电子邮件 |
| □学术会议 |
| □研讨会或工作坊 |
| □与利益相关者（研究的相关人员）的面对面会议 |
| □媒体采访或新闻发布会 |
| □新媒体（包括微信公众号、知乎、今日头条、B站、抖音等） |
| □技术标准/指南/规范 |
| □专利 |
| □其他，请注明： _________________* |

15. What methods do you usually use to disseminate research findings? [Multiple choice question] *

Please select all that apply.

| □Academic journals |
| --- |
| □Reports to funders |
| □Newsletter/Reports to department |
| □Policy briefs |
| □Targeted mailings |
| □Academic conferences |
| □Seminars/workshops |
| □Face-to-face meetings with stakeholders |
| □Media interviews/Press releases |
| □New media (e.g. WeChat Official Accounts, Zhihu, Bilibili, TikTok, etc.) |
| □Standards/guidelines |
| □Patent |
| □Other, please specify _________________* |

16. 在上述推广科研成果的渠道中，您认为哪种渠道对您的职业发展影响最大？ [多选题] *

可多选，请从15题的选项中选择影响最大的1-3项。

16. Of the methods above, which do you think generally have the most impact on your career trajectory? [Multiple choice question] *

Please select 1~3 options that apply from Question 15.

17. 在上述推广科研成果的渠道中，您认为哪种渠道对公共卫生实践或政策影响最大？ [多选题] *

可多选，请从15题的选项中选择影响最大的1-3项。

17. Of the methods above, which do you think generally have the most impact on public health practice or policy? [Multiple choice question] *

Please select 1~3 options that apply from Question 15.

18. 您为什么会推广您的科研成果？ [多选题] *

可多选，请选择所有符合的选项。

| □有助于提高社会各界对科研成果的认识 |
| --- |
| □有助于激发对科研成果的讨论或辩论 |
| □有助于影响政策 |
| □有助于影响实践 |
| □有助于证明科研经费的合理性 |
| □有助于今后进一步申请经费 |
| □有助于提高单位形象 |
| □有助于得到他人认可 |
| □有助于提高自己的沟通/传播能力 |
| □有助于满足岗位要求 |
| □出于个人兴趣 |
| □出于社会责任感 |
| □所在的学术圈有推广科研成果的良好氛围 |
| □其他，请注明 _________________* |

18. Why do you disseminate the findings of your research? [Multiple choice question] *

Please select all that apply.

| □To raise awareness of the findings |
| --- |
| □To stimulate discussion or debate |
| □To influence policy |
| □To influence practice |
| □To justify public funding |
| □To attract future funding |
| □To raise the organizational profile |
| □To be recognized and affirmed by others |
| □To improve your own communication |
| □To satisfy grant/contractual obligations |
| □Out of personal interest |
| □Out of social responsibility |
| □My own academic setting has a good atmosphere for dissemination |
| □Other, please specify _________________* |

      除向科研同仁推广科研成果外，将您的科研成果推广给**不从事科研工作、但同样需要了解和使用科研成果的其他人员或机构**也非常重要，我们将这类人员统称“非科研人员”。

      在接下来的调查中，我们将关注您**针对“非科研人员”的推广情况。**

      For the remainder of the survey, we ask that you focus specifically on the dissemination of research findings to **non-research audiences** such as public health practitioners, policymakers and/or other target populations.

19. 您向哪些机构和“非科研人员”（“非科研人员”指不从事科研工作的人员）推广过您的科研成果？ [多选题] *

可多选，请选择所有符合的选项。

| □医疗卫生机构及医务人员 |
| --- |
| □国家/地方卫健委（局） |
| □国家/地方疾控中心 |
| □人大代表或政协委员 |
| □非卫生系统政府机构 |
| □国际政府组织（如联合国机构） |
| □国际非政府组织 |
| □国内协会/学会 |
| □企业 |
| □经费支持机构 |
| □研究对象及其所在的社区 |
| □媒体 |
| □公众 |
| □其他，请注明 _________________* |

19. To which of the following non-research audiences have you directly disseminated the findings of your research? [Multiple choice question] *

Please select all that apply.

| □Health care providers |
| --- |
| □National/regional health departments |
| □National/regional center/agency for disease control and prevention |
| □Elected officials |
| □Non-health departments |
| □International governmental agencies |
| □International non-governmental organizations |
| □Academic society/association |
| □Enterprises |
| □Funding agencies |
| □The target population of your research |
| □The media |
| □The general public |
| □Other, please specify _________________* |

20. 您所在的单位是否支持您向“非科研人员”推广科研成果？ [单选题] *

| ○是 (请跳至第21题) |
| --- |
| ○否 (请跳至第22题) |
| ○不确定 (请跳至第22题) |

20. Is the dissemination of research findings to non-research audiences expected by your employer? [Single choice question] *

| ○Yes (Please skip to Question 21) |
| --- |
| ○No (Please skip to Question 22) |
| ○No (Please skip to Question 22) |

21. 您所在的单位如何评估您向“非科研人员”推广科研成果的情况？ [多选题] *

可多选，请选择所有符合的选项。

| □不评估 (请跳至第23题) |
| --- |
| □纳入绩效考核 (请跳至第23题) |
| □纳入职务/职称晋升要求 (请跳至第23题) |
| □其他，请注明 _________________* (请跳至第23题) |

21. How is your dissemination of research findings to non-research audiences evaluated by your employer? [Multiple choice question] *

Please select all that apply.

| □Not evaluated (Please skip to Question 23) |
| --- |
| □Performance assessment (Please skip to Question 23) |
| □Promotion/job requirements (Please skip to Question 23) |
| □Other, please specify _________________* (Please skip to Question 23) |

22. 您认为您所在的单位是否应该支持向“非科研人员”推广科研成果？ [单选题] *

| ○是 |
| --- |
| ○否 |
| ○不确定 |

22. Do you think the dissemination of research findings to non-research audiences should be expected by your employer? [Single choice question] *

| ○Yes |
| --- |
| ○No |
| ○Not sure |

23. 资助您科研经费的机构是否支持您向“非科研人员”推广科研成果？ [单选题] *

| ○是 (请跳至第24题) |
| --- |
| ○否 (请跳至第25题) |
| ○不确定 (请跳至第25题) |

23. Is the dissemination of research findings to non-research audiences expected by your funding agencies? [Single choice question] *

| ○Yes (Please skip to Question 24) |
| --- |
| ○No (Please skip to Question 25) |
| ○No (Please skip to Question 25) |

24. 资助您科研经费的机构如何评估您向“非科研人员”推广科研成果的情况？ [多选题] *

可多选，请选择所有符合的选项。

| □不评估 (请跳至第26题) |
| --- |
| □将推广情况作为任务合同书/考核指标的一部分 (请跳至第26题) |
| □其他，请注明 _________________* (请跳至第26题) |

24. How is your dissemination of research findings to non-research audiences evaluated by your funding agencies? [Multiple choice question] *

Please select all that apply.

| □Not evaluate (Please skip to Question 26) |
| --- |
| □As part of the task contract/assessment requirement (Please skip to Question 26) |
| □Other, please specify _________________* (Please skip to Question 26) |

25. 您认为资助您科研经费的机构是否应该支持向“非科研人员”推广科研成果？ [单选题] *

| ○是 |
| --- |
| ○否 |
| ○不确定 |

25. Do you think the dissemination of research findings to non-research audiences should be expected by your funding agencies? [Single choice question] *

| ○Yes |
| --- |
| ○No |
| ○Not sure |

26. 请给出您对以下信息的看法：[矩阵单选题] *

|  | 非常不同意 | 不同意 | 不确定 | 同意 | 非常同意 |
| --- | --- | --- | --- | --- | --- |
| 我向“非科研人员”推广科研成果所做出的努力在专业上获得了足够的认可。 | ○ | ○ | ○ | ○ | ○ |
| 向“非科研人员”推广科研成果对我的研究非常重要。 | ○ | ○ | ○ | ○ | ○ |
| 向“非科研人员”推广科研成果对我所在的部门/学系非常重要。 | ○ | ○ | ○ | ○ | ○ |

26. Please indicate your level of agreement with the following statement: [Single choice question] *

|  | Strongly disagree | Disagree | Neither agree or disagree | Agree | Strongly agree |
| --- | --- | --- | --- | --- | --- |
| I receive enough credit professionally for my efforts disseminating research findings to non-research audiences. | ○ | ○ | ○ | ○ | ○ |
| Dissemination to non-research audiences is important to my own research. | ○ | ○ | ○ | ○ | ○ |
| Dissemination to non-research audiences is important to the work of your unit/organization. | ○ | ○ | ○ | ○ | ○ |

27. 您认为以下哪些因素是向“非科研人员”推广科研成果的阻碍因素？ [多选题] *

可多选，请选择所有符合的选项。

| □不了解除了学术会议或发表期刊文章以外的推广途径 |
| --- |
| □不确定应该向哪些对象推广我的科研成果 |
| □不确定应该推广哪些内容 |
| □不确定推广会带来什么影响 |
| □缺乏推广科研成果的相关知识技能（如不了解如何从学术语言转化大众化语言等） |
| □缺乏时间和精力 |
| □未与利益相关者建立良好的联系 |
| □研究的时间安排里未考虑推广相关活动 |
| □对推广单项研究或阶段性科研成果不够有信心，比较谨慎甚至抗拒 |
| □所在的部门/学系/单位未将推广科研成果作为绩效考核内容之一 |
| □缺乏推广所需的经费支持 |
| □缺乏良好的合作机制 |
| □缺乏推广的平台 |
| □其他，请注明 _________________* |

27. What makes it difficult to disseminate your research findings to non-research audiences? [Multiple choice question] *

Please select all that apply.

| □Uncertainty on how best to disseminate beyond professional conferences/publications |
| --- |
| □Uncertainty about audience make-up |
| □Uncertainty about what to disseminate |
| □Uncertainty about the impact of dissemination |
| □Lack of knowledge and skills to disseminate (e.g. do not know how to transform academic language into language the target audiences prefer, etc.) |
| □Lack of time |
| □Lack of relationships with stakeholders |
| □Dissemination activities not in study timelines |
| □Hesitation/resistance to disseminate findings from a single study |
| □Lack of incentives |
| □Lack of financial resources for dissemination |
| □Lack of cooperation mechanisms |
| □Lack of platforms for dissemination |
| □Other, please specify _________________* |

28. 过去一年，您向“非科研人员”推广科研成果所花时间占全部科研时间的比例大约是？ [单选题] *

| ○无 |
| --- |
| ○少于5%（即每周少于2小时） |
| ○5%至10%之间 |
| ○10%至20%之间 |
| ○20%至30%之间 |
| ○30%至40%之间 |
| ○40%至50%之间 |
| ○多于50% |

28. Over the past year, please estimate the proportion of your own time that is dedicated to dissemination-related activities to non-research audiences? [Single choice question] *

| ○None |
| --- |
| ○Less than 5% (i.e., less than two hours a week) |
| ○Between 5% and 10% |
| ○Between 10% and 20% |
| ○Between 20% and 30% |
| ○Between 30% and 40% |
| ○Between 40% and 50% |
| ○More than 50% |

29. 您所在的部门/单位是否有专人或团队负责科研成果推广相关的活动？ [单选题] *

| ○是 (请跳至第30题) |
| --- |
| ○否 (请跳至第31题) |
| ○不确定 (请跳至第31题) |

29. Is there a dedicated person or team responsible for dissemination-related activities within your unit/organization? [Single choice question] *

| ○Yes (Please skip to Question 30) |
| --- |
| ○No (Please skip to Question 31) |
| ○Not sure (Please skip to Question 31) |

30. 该专人或团队设置在什么部门？ [单选题] *

| ○公关部门 (请跳至32题) |
| --- |
| ○宣传部门 (请跳至32题) |
| ○办公室/综合办公室 (请跳至32题) |
| ○事业发展部门 (请跳至32题) |
| ○转化处/转化办公室 (请跳至32题) |
| ○其他，请注明 _________________ * (请跳至32题) |

30. Where is this person or team housed within? [Single choice question] *

| ○Public relation office (Please skip to Question 32) |
| --- |
| ○Communication office (Please skip to Question 32) |
| ○General office (Please skip to Question 32) |
| ○Development office (Please skip to Question 32) |
| ○Translation office (Please skip to Question 32) |
| ○Other, please specify _________________ * (Please skip to Question 32) |

31. 您是否希望您所在的部门/单位有专人或团队来负责科研成果推广相关的活动？ [单选题] *

| ○是 |
| --- |
| ○否 |
| ○不确定 |

31. Would you like to have a dedicated person or team for dissemination-related activities? [Single choice question] *

| ○Yes |
| --- |
| ○No |
| ○Not sure |

32. 您所在的部门/学系/学院是否有推广科研成果的正式工作策略和计划？ [单选题] *

| ○是 |
| --- |
| ○否 |
| ○不确定 |

32. Does your unit/organization have a formal communication/dissemination strategy or plan? [Single choice question] *

| ○Yes |
| --- |
| ○No |
| ○Not sure |

33. 您是否使用理论、框架或模型来制定推广科研成果的计划？ [单选题] *

| ○总是 |
| --- |
| ○经常 |
| ○有时 |
| ○很少 |
| ○从不 (请跳至第35题) |
| ○不确定 (请跳至第35题) |
| ○我不负责制定推广相关活动计划 (请跳至第36题) |

33. How often have you used a framework or, theory, or logic model to plan dissemination-related activities? [Single choice question] *

| ○Always |
| --- |
| ○Usually |
| ○Sometimes |
| ○Rarely |
| ○Never (Please skip to Question 35) |
| ○Not sure (Please skip to Question 35) |
| ○I do not plan dissemination-related activities (Please skip to Question 36) |

34. 您使用什么理论、框架或模型来制定推广相关活动计划？ [多选题] *

可多选，请选择所有符合的选项。

| □创新扩散理论（Diffusion of Innovation） |
| --- |
| □兰德说服沟通模型（RAND Model of Persuasive Communication and Diffusion of Medical Innovation） |
| □有效推广模型（Effective Dissemination Strategies） |
| □研究成果转化本地化模型（Model for Locally Based Research Transfer Development） |
| □不知道 |
| □其他，请注明 _________________* |

34. What framework, theory, or logic model do you often used to plan dissemination-related activities [Multiple choice question] *

Please select all that apply.

| □Diffusion of Innovation |
| --- |
| □RAND Model of Persuasive Communication and Diffusion of Medical Innovation |
| □Effective Dissemination Strategies |
| □Model for Locally Based Research Transfer Development |
| □Do not know |
| □Other, please specify _________________* |

35. 您通常在研究过程中哪个阶段制定推广相关活动的计划？ [多选题] *

可多选，请选择所有符合的选项。

| □制定研究方案时（项目申请阶段） |
| --- |
| □数据收集或项目实施阶段 |
| □数据分析阶段 |
| □报告/文章撰写阶段 |
| □将科研成果发表在期刊上或在学术会议汇报之后 |
| □不确定 |

35. At what stage in the research process do you usually plan dissemination-related activities? [Multiple choice question] *

Please select all that apply

| □When the research is being formulated (at the proposal stage) |
| --- |
| □During the implementation/data collection stage |
| □During the data analysis stage |
| □At the draft report/manuscript stage |
| □After results have been published or presented at research meetings |
| □Not sure |

36. 您在研究过程中会经常邀请研究所涉及的利益相关者参与吗？ [单选题] *

“利益相关者”指与研究相关的人员或机构。

| ○总是 |
| --- |
| ○经常 |
| ○有时 |
| ○很少 |
| ○从不 (请跳至第38题) |
| ○不确定 (请跳至第38题) |

36. How often do you involve stakeholders (e.g., patients, community members, practitioners, policymakers) in your research progress? [Single choice question] *

| ○Always |
| --- |
| ○Usually |
| ○Sometimes |
| ○Rarely |
| ○Never (Please skip to Question 38) |
| ○Not sure (Please skip to Question 38) |

37. 您如何让利益相关者参与到您的研究和科研成果推广工作中？ [多选题] *

可多选，请选择所有符合的选项。

| □对利益相关者进行需求评估 |
| --- |
| □邀请利益相关者参加咨询委员会 |
| □邀请利益相关者参与用户测试 |
| □邀请利益相关者参与数据收集 |
| □请利益相关者提供相关证明或许可（如准许进入研究现场等） |
| □邀请利益相关者作为研究团队的正式成员 |
| □邀请利益相关者讨论研究结果 |
| □邀请利益相关者撰写研究报告或文章 |
| □通过利益相关者的社会关系网络推广科研成果 |
| □其他，请注明 _________________* |

37. How do you involve these stakeholder groups in your research and dissemination efforts? [Multiple choice question] *

Please select all that apply.

| □Assess stakeholders’ need |
| --- |
| □Engage stakeholders as advisors |
| □Engage stakeholders on user panels |
| □Engage stakeholders in data gathering |
| □Engage stakeholders to provide testimonials or endorsements |
| □Engage stakeholders as formal research team members |
| □Engage stakeholders to interpret data |
| □Engage stakeholders to write up findings |
| □Disseminate findings through stakeholders’ networks |
| □Other, please specify _________________* |

38. 您是否会针对某一类“非科研人员”撰写研究概要或主要科研成果报告？ [单选题] *

| ○总是 |
| --- |
| ○经常 |
| ○有时 |
| ○很少 |
| ○从不 |
| ○不确定 |

38. How often do you produce research summaries or key messages that are written for specific non-research audiences or groups? [Single choice question] *

| ○Always |
| --- |
| ○Usually |
| ○Sometimes |
| ○Rarely |
| ○Never |
| ○Not sure |

39. 您是否会对您的科研成果改变公共卫生实践或政策的情况进行评估？（如卫生健康部门使用您的科研成果或利用您的科研成果开展培训） [单选题] *

| ○总是 |
| --- |
| ○经常 |
| ○有时 |
| ○很少 |
| ○从不 (请跳至第41题) |
| ○不确定 (请跳至第41题) |

39. How often do you evaluate the uptake of your research in new settings, on changing public health practice or policy (e.g. use of your research by a health department, use of your research for the basis for training of practitioners)? [Single choice question] *

| ○Always |
| --- |
| ○Usually |
| ○Sometimes |
| ○Rarely |
| ○Never (Please skip to Question 41) |
| ○Not sure (Please skip to Question 41) |

40. 您采用什么方法来评估您的科研成果被采用的情况？ [多选题] *

可多选，请选择所有符合的选项。

| □调查 |
| --- |
| □定性方法 |
| □其他，请注明 _________________* |

40. What method(s) do you use to evaluate the uptake of your research in new settings? [Multiple choice question] *

Please select all that apply.

| □Surveys |
| --- |
| □Qualitative methods |
| □Other, please specify _________________* |

41. 总体而言，您如何评价您向“非科研人员”推广科研成果所做出的努力？ [单选题] *

| ○特别好 |
| --- |
| ○很好 |
| ○一般 |
| ○不好 |
| ○不确定 |

41. Overall, how do you rate your efforts to disseminate your research findings to non-research audiences? [Single choice question] *

| ○Excellent |
| --- |
| ○Good |
| ○Adequate |
| ○Poor |
| ○Not sure |

42. 请给出您对以下信息的看法：

科研人员有义务将他们的科研成果推广给那些需要了解和使用科研成果的人。 [单选题] *

| ○非常不同意 |
| --- |
| ○不同意 |
| ○不确定 |
| ○同意 |
| ○非常同意 |

42. Please indicate your level of agreement with the following statement:

It is an obligation of researchers to disseminate their research to those who need to learn about it and make use of the findings. [Single choice question] *

| ○Strongly disagree |
| --- |
| ○Disagree |
| ○Neither agree or disagree |
| ○Agree |
| ○Strongly agree |

您的答卷已经提交，感谢您的参与！

Thank you for taking the time to complete this questionnaire!
